# Prevalence of antibiotic use: a comparison across various European health care data sources

**DOI:** 10.1002/pds.3831

**Published:** 2015-07-07

**Authors:** Ruth Brauer, Ana Ruigómez, Gerry Downey, Andrew Bate, Luis Alberto Garcia Rodriguez, Consuelo Huerta, Miguel Gil, Francisco de Abajo, Gema Requena, Yolanda Alvarez, Jim Slattery, Mark de Groot, Patrick Souverein, Ulrik Hesse, Marietta Rottenkolber, Sven Schmiedl, Frank de Vries, Maurille Feudjo Tepie, Raymond Schlienger, Liam Smeeth, Ian Douglas, Robert Reynolds, Olaf Klungel

**Affiliations:** ^1^ Faculty of Epidemiology and Population HealthLondon School of Hygiene and Tropical MedicineLondonUK; ^2^ Fundación Centro Español de Investigación Farmacoepidemiológica (CEIFE)MadridSpain; ^3^ Amgen NVLondonUK; ^4^ EpidemiologyPfizer LtdSurreyUK; ^5^ Agencia Española de Medicamentos y Productos Sanitarios (AEMPS)MadridSpain; ^6^ Clinical Pharmacology UnitUniversity Hospital Príncipe de AsturiasMadridSpain; ^7^ Pharmacology Section, Department of Biomedical Sciences IIUniversity of Alcalá (UAH)MadridSpain; ^8^ European Medicines Agency (EMA)LondonUK; ^9^ Faculty of Science, Division of Pharmacoepidemiology and Clinical PharmacologyUtrecht UniversityUtrechtThe Netherlands; ^10^ Lægemiddelstyrelsen (Danish Medicines Agency)National Institute for Health Data and Disease ControlCopenhagenDenmark; ^11^ Institute of Medical Information Sciences, Biometry, and EpidemiologyLudwig‐Maximilians‐Universitaet MuenchenMunichGermany; ^12^ Department of Clinical Pharmacology, School of Medicine, Faculty of HealthWitten/Herdecke UniversityWittenGermany; ^13^ Philipp Klee‐Institute for Clinical PharmacologyHELIOS Clinic WuppertalWuppertalGermany; ^14^ MRC Lifecourse Epidemiology UnitSouthampton General HospitalSouthamptonUK; ^15^ School CAPHRI / Maastricht UniversityMaastrichtThe Netherlands; ^16^ Novartis Pharma AGBaselSwitzerland; ^17^ EpidemiologyPfizer Research & DevelopmentNew YorkNYUSA

**Keywords:** descriptive study, primary care databases, international comparisons, antibiotic agents, pharmacoepidemiology

## Abstract

**Purpose:**

There is widespread concern about increases in antibiotic use, but comparative data from different European countries on rates of use are lacking. This study was designed to measure and understand the variation in antibiotic utilization across five European countries.

**Methods:**

Seven European healthcare databases with access to primary care data from Denmark, Germany, the Netherlands, Spain and the UK were used to measure and compare the point and 1‐year‐period prevalence of antibiotic use between 2004 and 2009. Descriptive analyses were stratified by gender, age and type of antibiotic. Separate analyses were performed to measure the most common underlying indications leading to the prescription of an antibiotic.

**Results:**

The average yearly period prevalence of antibiotic use varied from 15 (Netherlands) to 30 (Spain) users per 100 patients. A higher prevalence of antibiotic use by female patients, the very young (0–9 years) and old (80+ years), was observed in all databases. The lowest point prevalence was recorded in June and September and ranged from 0.51 (Netherlands) to 1.47 (UK) per 100 patients per day. Twelve percent (Netherlands) to forty‐nine (Spain) percent of all users were diagnosed with a respiratory tract infection, and the most common type of antibiotic prescribed were penicillin.

**Conclusion:**

Using identical methodology in seven EU databases to assess antibiotic use allowed us to compare drug usage patterns across Europe. Our results contribute quantitatively to the true understanding of similarities and differences in the use of antibiotic agents in different EU countries. © 2015 The Authors. *Pharmacoepidemiology and Drug Safety* Published by John Wiley & Sons, Ltd.

## Introduction

Studies on antibiotic use in Europe have shown an increase in the total volume of outpatient antibiotic sales and reimbursements between 1997 and 2009.^1^, ^2^ There are few studies that have measured the prescription rates of antibiotic agents in different European countries using healthcare databases with access to primary care data, despite antibiotic agents being most commonly prescribed by general practitioners.^3^, ^4^ Whilst there has been an increase in the availability and use of electronic healthcare data sets for drug safety surveillance and pharmacoepidemiological hypothesis testing studies, using information from multiple databases for comparative purposes is challenging due to heterogeneity between databases in structure, terminology, underlying healthcare systems and data capture.^5^, ^6^, ^7^, ^8^, ^9^ Comparing the rates of antibiotic drug prescribing or dispensing between different countries using patient data as recorded in electronic healthcare data sets facilitates an understanding of the broad range of reported incidences of adverse events associated with the use of antibiotics, including hepatic injuries and antibiotic resistance.^10^, ^11^, ^12^, ^13^

The current study was designed to measure and understand the variation in antibiotic utilization across specific databases as part of ‘Pharmacoepidemiological Research on Outcomes of Therapeutics by a European Consortium’ (PROTECT).^14^ The primary objective of this study was to measure and to compare the point and 1‐year‐period prevalence of general practice antibiotic prescriptions or dispensations between 2004 and 2009 in five different European countries using seven healthcare databases. Additionally, this study aimed to describe the most common underlying diagnoses leading to the prescription of an antibiotic agent and the proportional use of different types of antibiotic agents per database and over time.

## Methods

### Data sources

The analyses were conducted in patient populations from the following databases: (i) the Clinical Practice Research Datalink (CPRD) from the UK; (ii) The Health Improvement Network (THIN) from the UK; (iii) the ‘Base de datos para la Investigación Farmacoepidemiologica en Atencion Primaria’ (BIFAP) — a Spanish computerized database of medical records of primary care; (iv) the Bavarian Association of Statutory Health Insurance Physicians (‘Kassenärztliche Vereinigung Bayerns’ [KVB]) database (henceforth referred to as the Bavarian Claims Database); (v) the Dutch Mondriaan Netherlands Primary Care Research Database (NPCRD); (vi) the Dutch Mondriaan Almere Healthcare group (AHC) database; and (vii) the Danish national registries.

The two UK general practice databases, CPRD and THIN, collect and archive the electronic medical records of more than 5 and 3 million active patients, respectively, covering over 8% of the UK population (Table 1).^5^, ^6^, ^15^ The UK databases overlap to some extent, but in 2012, they provided unique information for 268 and 168 practices, respectively.^16^ The BIFAP database, covering around 6.8% of the Spanish population, collects data from both GPs and paediatricians and currently has 1190 collaborating physicians from 9 different autonomous communities in Spain.^7^ Data from the Bavarian Claims Database are extracted from the Bavarian Association of Statutory Health Insurance Physicians accounting system.^17^ The German database includes population‐based data on diagnoses and medical services linked to outpatient treatment data through GPs and specialists. The Dutch Mondriaan project links data from various sources (pharmacy, GP and hospital pharmacy/laboratory). For the purposes of the current study, two different primary care databases within the Mondriaan project were used: the NPCRD and the AHC database.^9^ The Danish national registries, managed by the Danish Health and Medicines Authority (DKMA), contain information on hospital contacts, medication dispensing on a pharmacy level linked to individuals who redeemed the prescription, causes of death for the entire population and contact information of visits to GPs as well as specialists in private care.^18^

**Table 1:** Details of seven electronic health care databases with access to primary care data

Database	Country	Cumulative population number (2008)	Active population number (2008)	Start data availability	Data source	Coding diagnoses	Coding drugs	Exposure	Contribution
CPRD	UK	11 m	3.6 m	1987	GP	READ	BNF	Prescriptions	All
THIN	UK	7.8 m	3.1 m	2003	GP	READ	BNF	Prescriptions	No seasonality
BIFAP	Spain	3.2 m	1.6 m	2001	GP	ICPC	ATC	Prescriptions	All
Bavarian Claims Database	Germany	10.5 m	9.5 m	2001	Claims	ICD	ATC	Dispensations	No seasonality
Mondriaan NPCRD	The Netherlands	500 000	300 000	1991	GP	ICD/ICPC	ATC	Prescriptions	All
Mondriaan AHC	The Netherlands	200 000	150 000	1991	Multi‐source	ICD/ICPC	ATC	Dispensations	All
The Danish, DKMA managed, national databases	Denmark	5.2 m	5.2	1994 (medical product) 1979 (patient register)	Multi‐source	ICD	ATC	Dispensations	No prevalence by type

GP, General Practice; BNF, British National Formulary; ICPC, International Classification of Primary Care; ATC, Anatomical Therapeutic Chemical Classification System; ICD, International Classification of Diseases; m, million; CPRD, Clinical Practice Research Datalink; THIN, The Health Improvement Network; BIFAP, Base de datos para la Investigacion Farmacoepidemiologica en Atencion Primaria; NPCRD, Netherlands Primary Care Research Database; AHC, Almere Healthcare group; DKMA, Danish Health and Medicines Authority.

### Study population and period of valid data collection

The source population in all five countries included all individuals with access to primary health care.

As primary care is free at the point of delivery, it has almost universal coverage in the UK, Denmark, the Netherlands, Germany and Spain. Primary care physicians are gatekeepers to specialist care and are usually the first healthcare contact for all non‐emergency care. After registration, a general practice becomes the typical source of care. In most European countries, the choice of general practice is dependent on geographical proximity to the primary address of an individual.

The study population in every database was composed of all available patients of all ages with an active registration status during the study period of 1 January 2004 to 31 December 2009. In the Dutch Mondriaan AHC and the Bavarian Claims Database, the last available year of data collection was 2008. The remaining databases ended data collection in December 2009. Valid data collection started a year after the date when a practice became up to research quality standard or the date when a patient enrolled into a practice or the date that a practice was enrolled into the database, whichever came last. The follow‐up time of patients was censored on the date a patient died, the date a patient was transferred out of the practice, the end of the database's data collection or the date that the practice left the database, whichever came first.

### Exposure definition

Exposure was defined as a recorded prescription for an antibiotic agent listed in chapter 5 of the British National Formulary or therapeutic subgroup J01 of the Anatomical Therapeutic Chemical (ATC) Classification System. In the Danish, Bavarian and Mondriaan AHC databases, exposure was defined as the dispensing of an antibiotic agent to a registered individual. Prescriptions for topical antibiotics were excluded. Antibiotic agents were grouped in seven categories: tetracyclines, penicillins, cephalosporins, macrolides, aminoglycosides, quinolones and other antibiotics (sulphonamides and other combinations). To assess the point prevalence of antibiotic use, duration of exposure was calculated by dividing the total quantity of prescribed or dispensed antibiotics by the numeric daily dosage prescribed or dispensed. The mode (BIFAP only) or median duration of exposure to all antibiotic agents was imputed when information on the total quantity or the prescribed daily dosage was missing. The total number of patients registered in every database was based on the total active annual patient population at mid‐year from 2004 to 2009.

### Calculation of period and point prevalence

Annual 1‐year‐period prevalence was calculated by dividing the number of patients being prescribed an antibiotic in the study period of interest (numerator) by the number of patients available in every database at mid‐year (denominator). Antibiotic users were defined as patients with at least one recorded antibiotic prescription or dispensation. Crude prevalence rates and prevalence rates standardized by age and sex were calculated to account for potential differences in the age–sex distribution of the different populations across databases and over time. We applied direct standardization according to the 2008 European population standard.^19^ We quantified time trends in prevalence per database using Poisson regression. Separate analyses were performed to assess 1‐year‐period prevalence rates (2004–2009) of antibiotic drug use stratified by indication and gender in all databases. Indications for antibiotic agents were classified into the following three main groups: (i) respiratory infections; (ii) genitourinary tract infections; (iii) other infections; and (iv) unknown (for code lists, see Supporting Information). The latter category included patients with more than one of the aforementioned indications for the receipt of one antibiotic prescription. An indication for an antibiotic agent was assessed by searching for specific computer codes for infections within 2 weeks prior to or on the date of the first prescription in the year of interest. In the Bavarian Claims Database, diagnoses were linked to prescriptions if the prescription was dispensed in the quarter of the recorded underlying diagnosis. Additional free text information to link prescriptions to indications was only used in the BIFAP database. Separate analyses were performed to assess 1‐year‐period prevalence of antibiotic drug use stratified by major antibiotic groups and by yearly number of prescriptions per patient (1, 2–4, 5–10 and ≥11 prescriptions).

Point prevalence was assessed in the CPRD, BIFAP, DKMA and both Mondriaan databases on the 1st of March, June, September and December from 2004 to 2009 to take seasonal variations into account. For this analysis, antibiotic use was defined as having received a prescription/dispensation for an antibiotic agent before or on the 1st of March, June, September and December that lasted until or after the 1st of the same month.

## Results

### Overall comparison of antibiotic use

**Figure 1:**
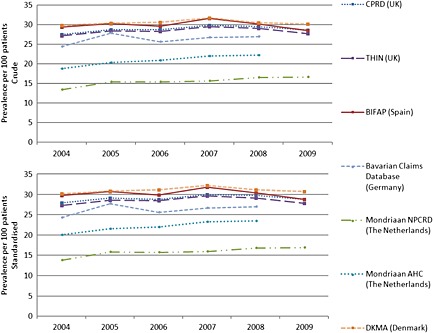
Comparison of the annual prevalence of antibiotic use (2004–2009) in seven European health care databases (crude and age/sex standardized). CPRD, Clinical Practice Research Datalink; THIN, The Health Improvement Network; BIFAP, Base de datos para la Investigacion Farmacoepidemiologica en Atencion Primaria; NPCRD, Netherlands Primary Care Research Database; AHC, Almere Healthcare group; DKMA, Danish Health and Medicines Authority

The highest crude prevalence of antibiotic use was observed in the BIFAP, DKMA, CPRD and THIN databases, with an average annual prevalence of 30 users per 100 patients in the Spanish and Danish databases and 29 users per 100 patients in the UK databases (Figure 1). The lowest annual prevalence of antibiotic use was observed in one of the Dutch databases (Mondriaan NPCRD: 15 users per 100 patients). When the prevalence of antibiotic use was adjusted for age and sex, the highest prevalence of antibiotic use was still observed in the Spanish and Danish databases (31 users per 100 patients), followed by the UK databases (29 users per 100 patients). The lowest adjusted annual prevalence of antibiotic use was observed in the Dutch Mondriaan NPCRD and AHC databases (16 and 22 users per 100 patients, respectively)

The yearly period prevalence of antibiotic users was relatively stable between 2004 and 2009. The results from the Poisson regression model showed no evidence of a clear time trend.

**Figure 2:**
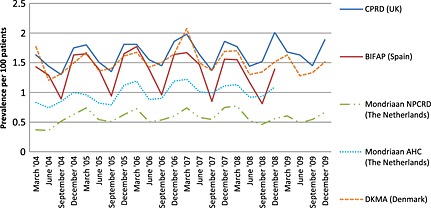
Point prevalence of antibiotic use (2004–2009) in five European health care databases. CPRD, Clinical Practice Research Datalink; BIFAP, Base de datos para la Investigacion Farmacoepidemiologica en Atencion Primaria; NPCRD, Netherlands Primary Care Research Database; AHC, Almere Healthcare group; DKMA, Danish Health and Medicines Authority

The lowest‐point prevalence of antibiotic use in the CPRD, BIFAP, Mondriaan and DKMA databases was recorded in the summer months (June and September). The seasonal difference in the number of prescriptions was more pronounced in BIFAP than in other databases, with an average increase of 42% in the proportion of patients who were prescribed an antibiotic in December and March compared with June and September (Figure 2).

### Comparison of the prevalence of antibiotic prescriptions by age

**Figure 3:**
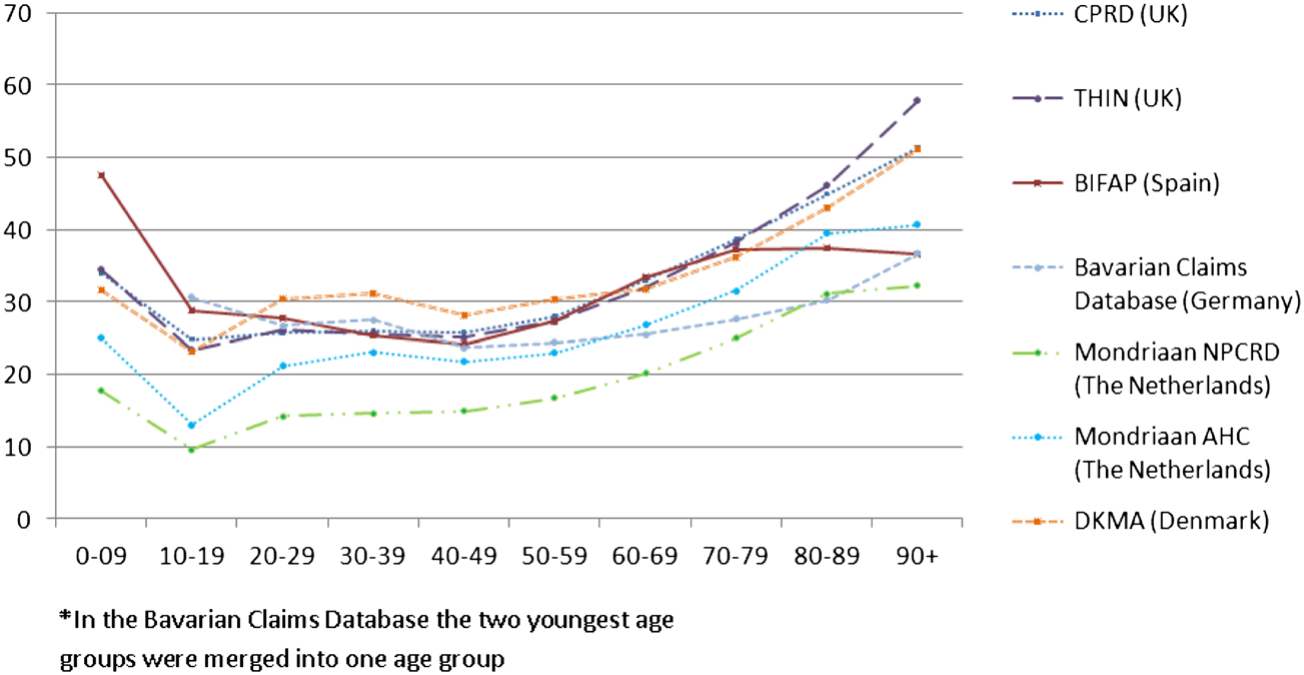
Comparison of 1‐year‐period prevalence of antibiotic use (2008) in seven European databases by age*. CPRD, Clinical Practice Research Datalink; THIN, The Health Improvement Network; BIFAP, Base de datos para la Investigacion Farmacoepidemiologica en Atencion Primaria; NPCRD, Netherlands Primary Care Research Database; AHC, Almere Healthcare group; DKMA, Danish Health and Medicines Authority

A higher annual prevalence of antibiotic use by female patients was observed in all databases (Table 2). Antibiotic use stratified by age groups showed a similar pattern in all seven databases. Antibiotic prescribing was highest in the very young (0–9 years) and very old (80+ years) patient populations and relatively stable from 10 to 59 years (Figure 3).

**Table 2:** Primary diagnosis of antibiotic users in five European databases stratified by gender (2008)

Database	Gender (ratio)	Respiratory tract infections	Genitourinary infections	Other infections	Unknown
CPRD					
	Males (1)	175 039 (35%)	13 508 (3%)	6415 (1%)	303 174 (61%)
	Females (1.5)	242 755 (33%)	58 416 (8%)	9078 (1%)	432 408 (58%)
THIN					
	Males (1)	153 679 (36%)	23 225 (5%)	14 870 (3%)	237 398 (55%)
	Females (1.49)	231 267 (35%)	105 685 (16%)	25 941 (4%)	288 780 (44%)
BIFAP					
	Males (1)	93 283 (52%)	8999 (5%)	15 311 (8%)	63 719 (35%)
	Females (1.19)	117 258 (46%)	35 333 (14%)	16 661 (7%)	83 986 (33%)
Bavarian Claims Database					
	Males (1)	599 615 (52%)	76 265 (7%)	87 727 (8%)	383 813 (33%)
	Females (1.24)	700 883 (42%)	305 663 (18%)	105 546 (6%)	553 215 (33%)
Mondriaan NPCRD					
	Males (1)	6292 (14%)	1323 (3%)	9827 (22%)	27 169 (61%)
	Females (1.57)	8030 (10%)	8016 (10%)	14 406 (18%)	48 485 (62%)
Mondriaan AHC					
	Males (1)	4455 (22%)	1090 (6%)	2930 (15%)	11 400 (57%)
	Females (1.52)	5918 (17%)	7557 (22%)	3746 (11%)	16 995 (50%)
DKMA					
	Males (1)	34 083 (11%)	15 819 (5%)	176 838 (55%)	92 957 (29%)
	Females (1.37)	42 754 (10%)	82 870 (19%)	202 871 (46%)	114 640 (26%)

CPRD, Clinical Practice Research Datalink; THIN, The Health Improvement Network; BIFAP, Base de datos para la Investigacion Farmacoepidemiologica en Atencion Primaria; NPCRD, Netherlands Primary Care Research Database; AHC, Almere Healthcare group; DKMA, Danish Health and Medicines Authority.

### Comparison of the prevalence of antibiotic use by underlying diagnoses

For a large proportion of antibiotic users, there were no known or multiple indications recorded within 2 weeks prior to or on the day of the first prescription. Of those patients for whom an indication was found in CPRD, THIN, BIFAP and the Bavarian Claims Database, a recording of a respiratory tract infection was the most common diagnosis (Table 2). In the Danish and Dutch Mondriaan NPCRD databases, most patients were diagnosed with an infection other than respiratory tract or genitourinary. In all databases, female antibiotic users were more often identified with a diagnosis of genitourinary tract infections than males.

### Comparison of the prevalence of antibiotic use by type and number of prescriptions

**Figure 4:**
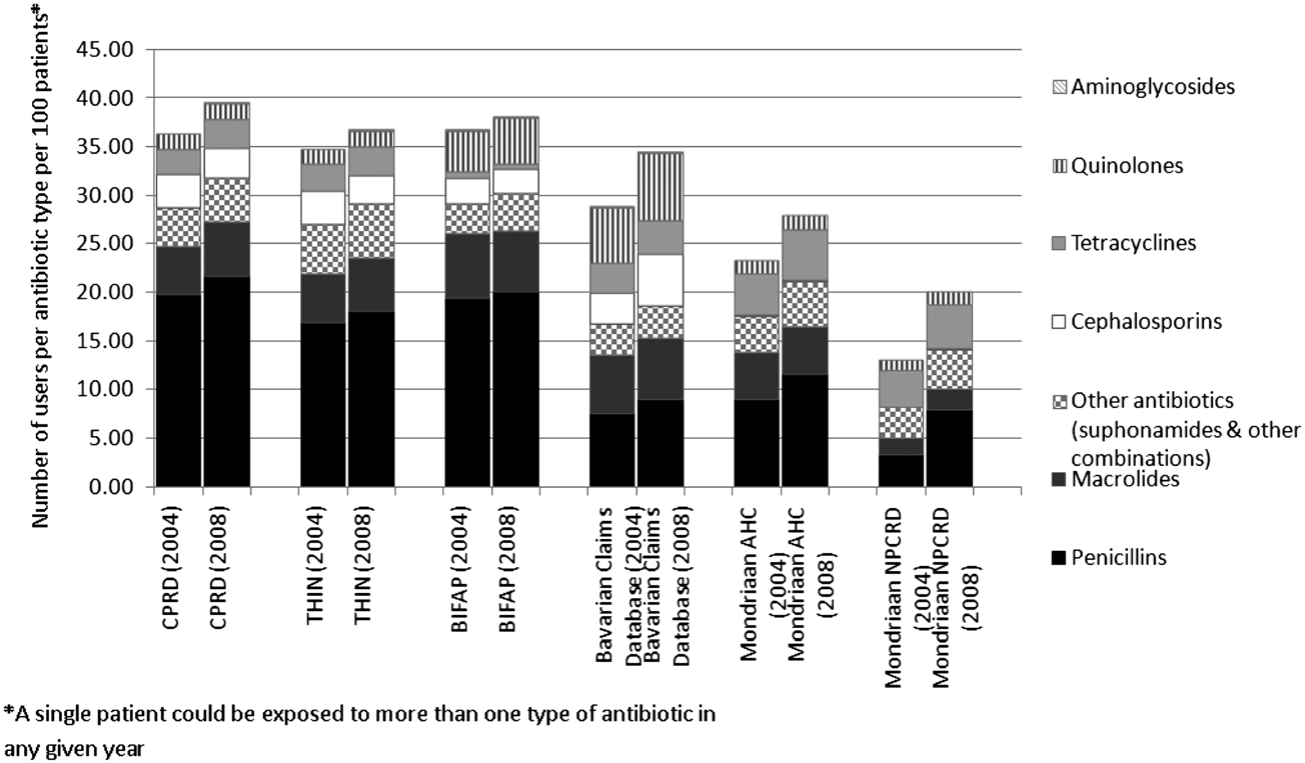
Proportional use of different types of antibiotic agents over time in six European databases in 2004 and 2008. CPRD, Clinical Practice Research Datalink; THIN, The Health Improvement Network; BIFAP, Base de datos para la Investigacion Farmacoepidemiologica en Atencion Primaria; NPCRD, Netherlands Primary Care Research Database; AHC, Almere Healthcare group; DKMA, Danish Health and Medicines Authority

The most common type of antibiotic drug class prescribed in every database was penicillins. Macrolides were the second most prescribed type of antibiotic agent in the British and Spanish databases. In the Dutch databases, the second most common class of antibiotics prescribed were tetracyclines. By comparison, the number of tetracyclines prescribed in the UK relative to the number of patients in the database was much smaller, whilst the relative number of cephalosporins prescribed was much higher than in the Dutch Mondriaan databases. In the German database, quinolones were the second most prescribed type of antibiotic class, whilst they were one of the least prescribed types of antibiotic in most other databases (Figure 4). In every database, the relative number of prescriptions for penicillins and quinolones was higher in 2008 compared with that in 2004. The use of tetracyclines and macrolides had also increased in almost every database, except BIFAP. Prescriptions for cephalosporins increased in the Dutch and German databases, but not in the other databases.

**Figure 5:**
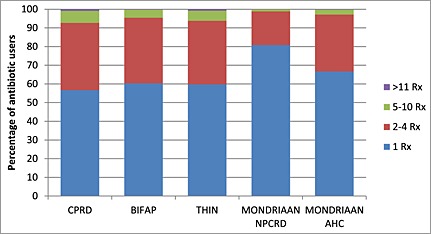
Proportional number of prescriptions/dispensings per individual across five databases in 2008. CPRD, Clinical Practice Research Datalink; THIN, The Health Improvement Network; BIFAP

Most antibiotic‐using patients, 81% in Mondriaan NPCRD, 67% in Mondriaan AHC, 57% in CPRD and 60% in THIN and BIFAP, received only one antibiotic prescription per year between 2004 and 2009. In Mondriaan NPCRD, 18% received two to four prescriptions per year, compared with 30% in Mondriaan AHC and 35% in the UK and Spanish databases. Very few patients (less than 3% in the Dutch databases and less than 6% in the UK and Spanish databases) received more than five prescriptions per year (Figure 5).

## Discussion

#### Summary

In this study, seven different European electronic healthcare databases with access to primary care data from Denmark, Germany, the Netherlands, Spain and the UK were used to compare the annual period prevalence of antibiotic use between 2004 and 2009. The yearly prevalence of crude and standardized antibiotic use was similar across databases, ranging from 26 (Germany) to 31 (Spain) users per 100 patients, with the exception of the Netherlands. The lowest average annual prevalence was observed in the Dutch Mondriaan NPCRD database: 16 users per 100 patients. There were similar patterns of antibiotic use across all databases with regard to gender, age and seasonality. Across all databases, most patients received only one prescription in any given year. National preferences for types of antibiotics prescribed, after penicillin, strongly differed per country.

Our results are similar to studies using sales and reimbursement data to compare rates of antibiotic use in Europe. When limited to the five countries that we included in our study, the European Surveillance of Antimicrobial Consumption (ESAC) project found the lowest antibiotic prescription rate in the Netherlands, followed by Germany. Danish and UK prescription rates were similar, and Spanish prescription rates were among the highest.^1^ The ESAC project reported the same antibiotic prescription trends over time. Our results are also in line with other studies that show there is more seasonal variability in antibiotic use in Spain compared with the UK and the Netherlands.^1^

#### Implications for research and practice

The implications for future pharmacoepidemiological research on antibiotics are twofold:

1. Our main findings show that the prevalence of antibiotic use in five European countries is very similar, with the exception of the Netherlands. The prevalence of antibiotic use in the Netherlands is not only lower compared with the prevalence in other countries, but most individuals that received an antibiotic agent only received a single antibiotic prescription in any given year (81%), which suggests that the prescribing behaviour of physicians is highly effective. Whilst we did not set out this descriptive study to find the best‐case scenario of antibiotic use, we believe that research using Dutch electronic health records could produce reliable estimates of the most conservative absolute estimates of adverse events associated with the use of antibiotics, including hepatic injuries and antibiotic resistance.
2. Whilst variability in the overall prevalence of antibiotic use was minimal across the UK, Spain, Denmark and Germany, there were differences in the recorded clinical indications for use and the intensity of use and clear differences in preferences for antibiotic classes between the countries. In studies investigating antibiotic‐induced injuries, the absence of recorded indications would restrict effective stratification by indication. Moreover, differences in preferences for individual antibiotic classes likely indicate significant differences in the composition of comparison groups.

#### Strengths and limitations

To our knowledge, this is the first study to measure and compare the prevalence of antibiotic use as recorded on a patient level in different European healthcare databases with access to primary care data. The primary strength of this study was the inclusion of the entire source population in every database. DKMA data (Denmark) represent complete quantitative national figures. Comparisons with national statistical data have shown that the CPRD, THIN and Mondriaan NPCRD databases are generalizable to the UK and Dutch populations, respectively.^20^, ^21^, ^22^ The data collected and archived in the BIFAP database are demographically representative of the Spanish population, whilst data are not collected from all autonomous communities in Spain. As the electronic primary care record is used to generate patient prescriptions in all participating countries, medication data are complete and accurate.

All seven partners used a single protocol and shared code lists to identify different types of antibiotic agents and underlying diagnoses. For some databases, dispensing records rather than prescription data were used, which may have affected the measured differences in prevalence of antibiotic use between the databases. For example, a small proportion of the dispensing data may have included antibiotic prescriptions prescribed by health care providers other than the regular primary care physicians, such as prescriptions received from dentists for emergency dental care. Different coding systems to measure prescriptions in every database (British National Formulary versus ATC codes) could also have caused some variability in the inclusion of some combinations of antibiotic agents.

We were not be able to detect patients who were prescribed or dispensed antibiotic agents outside primary care (i.e. in a hospital, by private doctors or by over‐the‐counter sales^24^, ^25^), and we were not able to confirm whether a patient actually took the drug. Therefore, the data may not represent the true use of antibiotics by the population. If information on duration of antibiotic use was missing, we imputed the median value of the duration of all other exposures, but acknowledge that this specific technique to impute missing values may have led to an underestimation or overestimation of duration of exposure in a very small group of antibiotic users. The point prevalence of antibiotic use may have been affected by some misclassification as for those patients with missing information the median duration of other prescriptions/dispensations was imputed. This may explain why in Figure 2, the recorded antibiotic use in CPRD is higher than in BIFAP, in contrast with Figure 1. Figure 2 is also affected by the choice of months to estimate seasonality. For instance, in Spain, the month with the lowest antibiotic consumption is August and the highest consumption is usually recorded in December, January or February, depending on the timing of flu epidemics.^25^

A limitation of the current study was that the use of antibiotic agents could not be directly linked to potential underlying diagnoses. In most databases, an indication for an antibiotic agent was assessed by computer search using specific codes, or free text when available, within 2 weeks prior to or on the date of the first prescription/dispensation in the year of interest. Prevalence of antibiotic use linked to underlying diagnoses was investigated in all seven databases using either International Classification of Diseases, International Classification of Primary Care or READ codes. Despite efforts to maximize comparability of the three different coding systems, there may have been some variability in the inclusion of underlying diagnoses. However, some of the differences in main indications for antibiotic use can be explained by differences in national guidelines for prescribing. For example, in the Netherlands, GPs are actively discouraged from prescribing antibiotic agents when patients experience uncomplicated respiratory tract infections, which may explain the low number of exposed patients with recorded respiratory tract infections in the Netherlands.^23^, ^24^

There were some differences in the annual prevalence of antibiotic agents between different databases within the same country (the UK and the Netherlands). The differences between the UK databases were very small. The differences in the Dutch data could be explained by the fact that the Mondriaan AHC data were collected in a single city (Almere) with a relatively young population, whereas the Mondriaan NPCRD comprises a national sample of GPs.

#### Conclusions

As healthcare databases are increasingly being used to conduct drug utilization studies and pharmacoepidemiological investigations, including studies that link the use of antibiotics to several health‐related outcomes, we believe that our results contribute quantitatively to the true understanding of the use of antibiotic agents in primary care in different EU countries.

## Conflict of Interest

A. B., R. S. and R. R. belong to EFPIA (European Federation of Pharmaceutical Industries and Association) member companies in the IMI JU and costs related to their part in the research were carried by the respective company as in‐kind contribution under the IMI JU scheme. The views expressed are those of the authors only and not of their respective institution or company. None of the results presented in the current manuscript were published elsewhere.

Key Points

- Despite heterogeneity between seven European electronic health care databases in structure, terminology, underlying healthcare systems and data capture, we found relatively stable and similar yearly prevalence rates of crude and standardized antibiotic use between 2004 and 2009, with the exception of the Netherlands.
- There were clear differences in preference for antibiotic classes, recorded clinical indications for use and intensity of use between the five European countries in this study, which has implications for comparative studies investigating antibiotic‐induced injuries.
- The low prevalence rate of antibiotic use in the Netherlands in combination with the low intensity of use suggests that Dutch electronic health records could produce reliable estimates of the most conservative absolute estimates of adverse events associated with the use of antibiotics.

## Ethics Statement

The protocol was approved by the Spanish Agency for Medical products (AEMPS) to be performed in the BIFAP database as part of the PROTECT project and was registered in the ENCePP electronic register of studies (http://www.encepp.eu/encepp/studiesDatabase.jsp). CPRD ISAC approval for the study was obtained in October 2011 (11_019A_2). We maintained a blinding procedure until the final results from both CPRD and BIFAP were submitted to the coordinating centre at Utrecht University.

## Supporting information

Supporting info item
