# Supplementary material for: Prevalence of antibiotic use: a comparison across various European health care data sources
Source: Pharmacoepidemiol Drug Saf. 2015 Jul 7;25(Suppl Suppl 1):11–20. doi: 10.1002/pds.3831 (PMC4918309; doi:10.1002/pds.3831)
Supplement: Supplementary file 1 — Supporting info item [file PDS-25-11-s001.docx]

**Supplementary material**

**Code lists for Antibiotic indications**

**Respiratory / ORL infections**

ICPC Codes Description

H70 Otitis externa

H71 Acute otitis media/myringitis

H72 Serous otitis media

H73 Eustachian salpingitis

H74 Chronic otitis media

R71 Whooping cough

R72 Strep throat

R73 Boil/abscess nose

R74 Upper respiratory infection acute

R75 Sinusitis acute/chronic

R76 Tonsillitis acute

R77 Laryngitis/tracheitis acute

R78 Acute bronchitis/bronchiolitis

R79 Chronic bronchitis

R80 Influenza

R81 Pneumonia

R82 Pleurisy/pleural effusion

R83 Respiratory infection other

R84 Malignant neoplasm bronchus/lung

A70 Tuberculosis

Read Codes Description

1414.00 H/O: scarlatina

1419.00 H/O: pertussis

1419.11 H/O: whooping cough

141D.00 H/O: diphtheria

14B2.00 H/O: pneumonia

14B3.11 H/O: bronchitis

1713.00 Productive cough -clear sputum

1714.00 Productive cough -green sputum

1715.00 Productive cough-yellow sputum

1716.00 Productive cough NOS

171F.00 Cough with fever

171G.00 Bovine cough

176..00 C/O - catarrh

1761.00 C/O bronchial catarrh

2524.11 O/E - cold sore

2D24.00 O/E -nasal disch.-mucopurulent

2DA2.00 O/E-maxillary sinus tenderness

2DA3.00 O/E - frontal sinus tenderness

2DA4.00 O/E - maxillary transilluminat

2DB..11 O/E - tonsils enlarged

2DB2.00 O/E - tonsils hyperaemic

2DB3.00 O/E - tonsils mod. enlarged

2DB4.00 O/E - tonsils grossly enlarged

2DB5.00 O/E - tonsils - quinsy present

2DB5.11 O/E - quinsy present

2DB6.00 O/E - follicular tonsillitis

2DB7.00 O/E - exudate on tonsils

2DC1.00 O/E - pharynx hyperaemic

2DC1.11 O/E - fauces injected

2DC2.00 O/E - granular pharyngitis

2DC3.00 Inflamed throat

3317100 Dick test positive

3318100 Schick test positive

3325.00 Mantoux: positive

4D23.00 Pleural fluid purulent

4E23.00 Sputum: mucopurulent

4E28.00 Yellow sputum

4E29.00 Green sputum

4E36.00 Sputum: pus cells present

4E37.00 Sputum: organism on gram stain

4E38.00 Sputum: tubercle on Z-N stain

4E38.11 Acid fast bacilli in sputum

4E39.00 Microscopy (acid fast bacilli)

4E3Z.11 Sputum evidence of infection

4JF2000 Nose swab culture positive

4JF4000 Throat swab culture positive

65V7.00 Notification of scarlet fever

65VA.00 Notification of whooping cough

A022200 Salmonella pneumonia

A054.00 Amoebic lung abscess

A101.00 Tuberculous pleurisy in primary progressive tuberculosis

A10z.00 Primary tuberculous infection NOS

A11..00 Pulmonary tuberculosis

A11..11 Lung tuberculosis

A110.00 Infiltrative lung tuberculosis

A111.00 Nodular lung tuberculosis

A112.00 Tuberculosis of lung with cavitation

A113.00 Tuberculosis of bronchus

A114.00 Tuberculous fibrosis of lung

A115.00 Tuberculous bronchiectasis

A116.00 Tuberculous pneumonia

A117.00 Tuberculous pneumothorax

A11y.00 Other specified pulmonary tuberculosis

A11z.00 Pulmonary tuberculosis NOS

A12..00 Other respiratory tuberculosis

A120.00 Tuberculous pleurisy

A120000 Tuberculosis of pleura

A120100 Tuberculous empyema

A120200 Tuberculous hydrothorax

A120z00 Tuberculous pleurisy NOS

A122.00 Isolated tracheal or bronchial tuberculosis

A122000 Isolated tracheal tuberculosis

A122100 Isolated bronchial tuberculosis

A122z00 Isolated tracheal or bronchial tuberculosis NOS

A123.00 Tuberculous laryngitis

A124.00 Resp TB bacteriologically and histologically confirmed

A124000 TB lung confirm sputum microscopy with or without culture

A124100 Tuberculosis of lung, confirmed by culture only

A124200 Tuberculosis of lung, confirmed histologically

A124300 Tuberculosis of lung, confirmed by unspecified means

A124500 Tuberculosis of larynx, trachea & bronchus conf bact/hist'y

A124600 Tuberculous pleurisy, conf bacteriologically/histologically

A124700 Primary respiratory TB confirm bact and histologically

A125000 Tuberculosis of lung, bacteriologically & histolog'y neg

A125100 Tuberculosis lung bact and histological examin not done

A125200 Prim respiratory TB without mention of bact or hist confirm

A125X00 Resp TB unspcf,w'out mention/bacterial or histol confrmtn

A12y.00 Other specified respiratory tuberculosis

A12y100 Tuberculosis of nasopharynx

A12y200 Tuberculosis of nasal septum

A12y300 Tuberculosis of nasal sinus

A12yz00 Other specified respiratory tuberculosis NOS

A203.00 Primary pneumonic plague

A204.00 Secondary pneumonic plague

A205.00 Pneumonic plague, unspecified

A211000 Cryptogenic tularaemia

A212.00 Pulmonary tularaemia

A221.00 Pulmonary anthrax

A310.00 Pulmonary mycobacterial infection

A310.11 Battey disease

A310000 Pulmonary mycobacterium avium-intracellulare infection

A320.00 Faucial diphtheria

A321.00 Nasopharyngeal diphtheria

A322.00 Anterior nasal diphtheria

A323.00 Laryngeal diphtheria

A33..00 Whooping cough

A33y.00 Whooping cough - other specified organism

A33y000 Bordetella bronchiseptica

A33yz00 Other whooping cough NOS

A33z.00 Whooping cough NOS

A34..00 Streptococcal sore throat and scarlatina

A340.00 Streptococcal sore throat

A340000 Streptococcal angina

A340100 Streptococcal laryngitis

A340200 Streptococcal pharyngitis

A340300 Streptococcal tonsillitis

A340z00 Streptococcal sore throat NOS

A341.00 Scarlet fever - scarlatina

A341.11 Scarlet fever

A341.12 Scarlatina

A34z.00 Streptococcal sore throat with scarlatina NOS

A383000 Fusobacterial necrotising tonsillitis

A391.00 Pulmonary actinomycosis

A39y000 Pulmonary nocardiosis

A3A1.00 Rhinoscleroma

A3A2.00 Whipple's disease

A3By100 Eaton's agent infection

A3By400 Pleuropneumonia-like organism (PPLO) infection

A521.00 Varicella pneumonitis

A542.11 Cold sore (herpetic)

A54x400 Herpes simplex pneumonia

A551.00 Postmeasles pneumonia

A730.00 Ornithosis with pneumonia

A741.00 Epidemic pleurodynia

A741.11 Bornholm disease

A741.12 Devil's grip

A785000 Cytomegaloviral pneumonitis

A78A100 Chlamydial infection of pharynx

A79A.00 Respiratory syncytial virus infection

A7y0200 Resp syncytial virus as cause of dis class to other chapters

A912300 Primary tonsil syphilis

A913200 Secondary syphilis of pharynx

A951.00 Syphilis of lung

A986.00 Gonococcal pharynx infection

AA12.00 Vincent's pharyngitis

AA1z.11 Vincent's laryngitis

AA1z.12 Vincent's tonsillitis

AA25.11 Rhinopharyngitis mutilans

AB24.00 Candidiasis of lung

AB24.11 Pneumonia - candidal

AB2y111 Otomycosis in moniliasis

AB30.00 Primary pulmonary coccidioidomycosis

AB30000 Acute pulmonary coccidioidomycosis

AB30100 Chronic pulmonary coccidioidomycosis

AB40500 Histoplasma capsulatum with pneumonia

AB40600 Acute pulmonary histoplasmosis capsulati

AB40700 Chronic pulmonary histoplasmosis capsulati

AB41500 Histoplasma duboisii with pneumonia

AB42.00 Pulmonary histoplasmosis

AB4z500 Histoplasmosis with pneumonia

AB50100 Primary pulmonary blastomycosis

AB50400 Acute pulmonary blastomycosis

AB50500 Chronic pulmonary blastomycosis

AB51100 Pulmonary paracoccidioidomycosis

AB60.00 Rhinosporidiosis

AB61300 Pulmonary sporotrichosis

AB63.00 Aspergillosis

AB63000 Invasive pulmonary aspergillosis

AB63100 Tonsillar aspergillosis

AB63400 Pulmonary aspergillus disease

AB63X00 Aspergillosis, unspecified

AB64.00 Mycotic mycetomas

AB64000 Actinomycetoma

AB65000 Pulmonary cryptococcosis

AB67000 Pulmonary mucormycosis

AC0..11 Schistosomiasis - pulmonary

AC12.11 Lung fluke disease

AC21.00 Lung echinococcus granulosus

AD04.00 Toxoplasma pneumonitis

AD40300 Nasopharyngeal myiasis

AE00.00 Late effects of respiratory tuberculosis

Ayu1000 [X]Other resp tubercul,confirmd bacteriologicly+histologicly

Ayu1100 [X]Resp tuberculos unspcfd,confirmd bacteriolog+histologicly

Ayu1200 [X]Oth resp tubercul, w'out m/bacteriol or histol confirmatn

Ayu1300 [X]Resp TB unspcf,w'out mention/bacterial or histol confrmtn

Ayu3900 [X]Whooping cough due to other Bordetella species

Ayu3A00 [X]Whooping cough, unspecified

Ayu5500 [X]Other Vincent's infections

AyuER00 [X]Mycetoma, unspecified

AyuET00 [X]Pulmonary histoplasmosis capsulati, unspecified

AyuEU00 [X]Other pulmonary aspergillosis

AyuJ400 [X]Sequelae of respiratory and unspecified tuberculosis

AyuKN00 [X]Resp syncytial virus/cause/diseases classfd/oth chapters

H0...00 Acute respiratory infections

H00..00 Acute nasopharyngitis

H00..11 Common cold

H00..12 Coryza - acute

H00..13 Febrile cold

H00..14 Nasal catarrh - acute

H00..15 Pyrexial cold

H00..16 Rhinitis - acute

H01..00 Acute sinusitis

H01..11 Sinusitis

H010.00 Acute maxillary sinusitis

H010.11 Antritis - acute

H011.00 Acute frontal sinusitis

H012.00 Acute ethmoidal sinusitis

H013.00 Acute sphenoidal sinusitis

H01y.00 Other acute sinusitis

H01y000 Acute pansinusitis

H01yz00 Other acute sinusitis NOS

H01z.00 Acute sinusitis NOS

H02..00 Acute pharyngitis

H02..11 Sore throat NOS

H02..12 Viral sore throat NOS

H02..13 Throat infection - pharyngitis

H020.00 Acute gangrenous pharyngitis

H021.00 Acute phlegmonous pharyngitis

H022.00 Acute ulcerative pharyngitis

H023.00 Acute bacterial pharyngitis

H023000 Acute pneumococcal pharyngitis

H023100 Acute staphylococcal pharyngitis

H023z00 Acute bacterial pharyngitis NOS

H024.00 Acute viral pharyngitis

H025.00 Allergic pharyngitis

H02z.00 Acute pharyngitis NOS

H03..00 Acute tonsillitis

H03..11 Throat infection - tonsillitis

H03..12 Tonsillitis

H030.00 Acute erythematous tonsillitis

H031.00 Acute follicular tonsillitis

H032.00 Acute ulcerative tonsillitis

H033.00 Acute catarrhal tonsillitis

H034.00 Acute gangrenous tonsillitis

H035.00 Acute bacterial tonsillitis

H035000 Acute pneumococcal tonsillitis

H035100 Acute staphylococcal tonsillitis

H035z00 Acute bacterial tonsillitis NOS

H036.00 Acute viral tonsillitis

H037.00 Recurrent acute tonsillitis

H03z.00 Acute tonsillitis NOS

H04..00 Acute laryngitis and tracheitis

H040.00 Acute laryngitis

H040000 Acute oedematous laryngitis

H040100 Acute ulcerative laryngitis

H040200 Acute catarrhal laryngitis

H040300 Acute phlegmonous laryngitis

H040400 Acute haemophilus influenzae laryngitis

H040500 Acute pneumococcal laryngitis

H040600 Acute suppurative laryngitis

H040w00 Acute viral laryngitis unspecified

H040x00 Acute bacterial laryngitis unspecified

H040z00 Acute laryngitis NOS

H041.00 Acute tracheitis

H041000 Acute tracheitis without obstruction

H041100 Acute tracheitis with obstruction

H041z00 Acute tracheitis NOS

H042.00 Acute laryngotracheitis

H042.11 Laryngotracheitis

H042000 Acute laryngotracheitis without obstruction

H042100 Acute laryngotracheitis with obstruction

H042z00 Acute laryngotracheitis NOS

H043.00 Acute epiglottitis (non strep)

H043.11 Viral epiglottitis

H043000 Acute epiglottitis without obstruction

H043100 Acute epiglottitis with obstruction

H043z00 Acute epiglottitis NOS

H044.00 Croup

H04z.00 Acute laryngitis and tracheitis NOS

H05..00 Other acute upper respiratory infections

H050.00 Acute laryngopharyngitis

H051.00 Acute upper respiratory tract infection

H052.00 Pharyngotracheitis

H053.00 Tracheopharyngitis

H054.00 Recurrent upper respiratory tract infection

H055.00 Pharyngolaryngitis

H05y.00 Other upper respiratory infections of multiple sites

H05z.00 Upper respiratory infection NOS

H05z.11 Upper respiratory tract infection NOS

H05z.12 Viral upper respiratory tract infection NOS

H06..00 Acute bronchitis and bronchiolitis

H060.00 Acute bronchitis

H060.11 Acute wheezy bronchitis

H060000 Acute fibrinous bronchitis

H060100 Acute membranous bronchitis

H060200 Acute pseudomembranous bronchitis

H060300 Acute purulent bronchitis

H060400 Acute croupous bronchitis

H060500 Acute tracheobronchitis

H060600 Acute pneumococcal bronchitis

H060700 Acute streptococcal bronchitis

H060800 Acute haemophilus influenzae bronchitis

H060900 Acute neisseria catarrhalis bronchitis

H060A00 Acute bronchitis due to mycoplasma pneumoniae

H060B00 Acute bronchitis due to coxsackievirus

H060C00 Acute bronchitis due to parainfluenza virus

H060D00 Acute bronchitis due to respiratory syncytial virus

H060E00 Acute bronchitis due to rhinovirus

H060F00 Acute bronchitis due to echovirus

H060v00 Subacute bronchitis unspecified

H060w00 Acute viral bronchitis unspecified

H060x00 Acute bacterial bronchitis unspecified

H060z00 Acute bronchitis NOS

H061.00 Acute bronchiolitis

H061000 Acute capillary bronchiolitis

H061100 Acute obliterating bronchiolitis

H061200 Acute bronchiolitis with bronchospasm

H061300 Acute exudative bronchiolitis

H061400 Obliterating fibrous bronchiolitis

H061500 Acute bronchiolitis due to respiratory syncytial virus

H061600 Acute bronchiolitis due to other specified organisms

H061z00 Acute bronchiolitis NOS

H062.00 Acute lower respiratory tract infection

H06z.00 Acute bronchitis or bronchiolitis NOS

H06z000 Chest infection NOS

H06z011 Chest infection

H06z100 Lower resp tract infection

H06z111 Respiratory tract infection

H06z112 Acute lower respiratory tract infection

H06z200 Recurrent chest infection

H07..00 Chest cold

H0y..00 Other specified acute respiratory infections

H0z..00 Acute respiratory infection NOS

H120.11 Catarrh unspecified

H120100 Chronic catarrhal rhinitis

H120111 Catarrhal child

H120400 Chronic infective rhinitis

H120500 Chronic ulcerative rhinitis

H120600 Chronic membranous rhinitis

H120700 Chronic fibrinous rhinitis

H120z00 Chronic rhinitis NOS

H121200 Granular pharyngitis

H121600 Chronic follicular pharyngitis

H121z00 Chronic pharyngitis NOS

H122.00 Chronic nasopharyngitis

H12z.00 Chronic pharyngitis and nasopharyngitis NOS

H13..00 Chronic sinusitis

H13..11 Chronic rhinosinusitis

H130.00 Chronic maxillary sinusitis

H130.11 Antritis - chronic

H130.12 Maxillary sinusitis

H131.00 Chronic frontal sinusitis

H131.11 Frontal sinusitis

H132.00 Chronic ethmoidal sinusitis

H133.00 Chronic sphenoidal sinusitis

H134.00 Fistula of nasal sinus

H135.00 Recurrent sinusitis

H13y.00 Other chronic sinusitis

H13y000 Chronic pansinusitis

H13y100 Pansinusitis

H13yz00 Other chronic sinusitis NOS

H13z.00 Chronic sinusitis NOS

H14..00 Chronic tonsil and adenoid disease

H14..11 Adenoid disease - chronic

H14..12 Tonsil disease - chronic

H140.00 Chronic tonsillitis

H140.11 Chronic adenoiditis

H143.00 Chronic adenotonsillitis

H14y600 Lingular tonsillitis

H15..00 Peritonsillar abscess - quinsy

H15..11 Quinsy

H16..00 Chronic laryngitis and laryngotracheitis

H160.00 Chronic laryngitis

H160000 Chronic simple laryngitis

H160100 Chronic catarrhal laryngitis

H160z00 Chronic laryngitis NOS

H161.00 Chronic laryngotracheitis

H16z.00 Chronic laryngitis NOS

H1y1.12 Nasal vestibulitis

H1y1000 Nasal septum abscess

H1y1700 Nasal septal granuloma

H1y1z14 Nasal infection

H1y2100 Pharynx or nasopharynx cellulitis

H1y2200 Parapharyngeal abscess

H1y2300 Retropharyngeal abscess

H1y2600 Pharynx or nasopharynx abscess

H1y5000 Abscess of vocal cords

H1y5100 Cellulitis of vocal cords

H1y5200 Granuloma of vocal cords

H1y7100 Cellulitis of larynx

H1y7500 Abscess of larynx

H1yz000 Abscess of trachea

H2...00 Pneumonia and influenza

H20..00 Viral pneumonia

H20..11 Chest infection - viral pneumonia

H200.00 Pneumonia due to adenovirus

H201.00 Pneumonia due to respiratory syncytial virus

H202.00 Pneumonia due to parainfluenza virus

H20y.00 Viral pneumonia NEC

H20z.00 Viral pneumonia NOS

H21..00 Lobar (pneumococcal) pneumonia

H21..11 Chest infection - pneumococcal pneumonia

H22..00 Other bacterial pneumonia

H22..11 Chest infection - other bacterial pneumonia

H220.00 Pneumonia due to klebsiella pneumoniae

H221.00 Pneumonia due to pseudomonas

H222.00 Pneumonia due to haemophilus influenzae

H222.11 Pneumonia due to haemophilus influenzae

H223.00 Pneumonia due to streptococcus

H223000 Pneumonia due to streptococcus, group B

H224.00 Pneumonia due to staphylococcus

H22y.00 Pneumonia due to other specified bacteria

H22y000 Pneumonia due to escherichia coli

H22y011 E.coli pneumonia

H22y100 Pneumonia due to proteus

H22y200 Pneumonia - Legionella

H22yX00 Pneumonia due to other aerobic gram-negative bacteria

H22yz00 Pneumonia due to bacteria NOS

H22z.00 Bacterial pneumonia NOS

H23..00 Pneumonia due to other specified organisms

H23..11 Chest infection - pneumonia organism OS

H230.00 Pneumonia due to Eaton's agent

H231.00 Pneumonia due to mycoplasma pneumoniae

H232.00 Pneumonia due to pleuropneumonia like organisms

H233.00 Chlamydial pneumonia

H23z.00 Pneumonia due to specified organism NOS

H24..00 Pneumonia with infectious diseases EC

H24..11 Chest infection with infectious disease EC

H240.00 Pneumonia with measles

H241.00 Pneumonia with cytomegalic inclusion disease

H242.00 Pneumonia with ornithosis

H243.00 Pneumonia with whooping cough

H243.11 Pneumonia with pertussis

H244.00 Pneumonia with tularaemia

H245.00 Pneumonia with anthrax

H246.00 Pneumonia with aspergillosis

H247.00 Pneumonia with other systemic mycoses

H247000 Pneumonia with candidiasis

H247100 Pneumonia with coccidioidomycosis

H247200 Pneumonia with histoplasmosis

H247z00 Pneumonia with systemic mycosis NOS

H24y.00 Pneumonia with other infectious diseases EC

H24y000 Pneumonia with actinomycosis

H24y100 Pneumonia with nocardiasis

H24y200 Pneumonia with pneumocystis carinii

H24y300 Pneumonia with Q-fever

H24y400 Pneumonia with salmonellosis

H24y500 Pneumonia with toxoplasmosis

H24y600 Pneumonia with typhoid fever

H24y700 Pneumonia with varicella

H24yz00 Pneumonia with other infectious diseases EC NOS

H24z.00 Pneumonia with infectious diseases EC NOS

H25..00 Bronchopneumonia due to unspecified organism

H25..11 Chest infection - unspecified bronchopneumonia

H26..00 Pneumonia due to unspecified organism

H26..11 Chest infection - pnemonia due to unspecified organism

H260.00 Lobar pneumonia due to unspecified organism

H260000 Lung consolidation

H261.00 Basal pneumonia due to unspecified organism

H262.00 Postoperative pneumonia

H27..00 Influenza

H270.00 Influenza with pneumonia

H270.11 Chest infection - influenza with pneumonia

H270000 Influenza with bronchopneumonia

H270100 Influenza with pneumonia, influenza virus identified

H270z00 Influenza with pneumonia NOS

H271.00 Influenza with other respiratory manifestation

H271000 Influenza with laryngitis

H271100 Influenza with pharyngitis

H271z00 Influenza with respiratory manifestations NOS

H27y.00 Influenza with other manifestations

H28..00 Atypical pneumonia

H30..00 Bronchitis unspecified

H30..11 Chest infection - unspecified bronchitis

H30..12 Recurrent wheezy bronchitis

H300.00 Tracheobronchitis NOS

H301.00 Laryngotracheobronchitis

H302.00 Wheezy bronchitis

H30z.00 Bronchitis NOS

H31..00 Chronic bronchitis

H310.00 Simple chronic bronchitis

H310000 Chronic catarrhal bronchitis

H311.00 Mucopurulent chronic bronchitis

H311000 Purulent chronic bronchitis

H311100 Fetid chronic bronchitis

H311z00 Mucopurulent chronic bronchitis NOS

H312200 Acute exacerbation of chronic obstructive airways disease

H313.00 Mixed simple and mucopurulent chronic bronchitis

H31y.00 Other chronic bronchitis

H31y000 Chronic tracheitis

H31y100 Chronic tracheobronchitis

H31yz00 Other chronic bronchitis NOS

H31z.00 Chronic bronchitis NOS

H341.00 Post-infective bronchiectasis

H3y0.00 Chronic obstruct pulmonary dis with acute lower resp infectn

H450.00 Pneumoconiosis associated with tuberculosis

H50..00 Empyema

H500.00 Empyema with fistula

H500000 Empyema with bronchocutaneous fistula

H500100 Empyema with bronchopleural fistula

H500200 Empyema with hepatopleural fistula

H500300 Empyema with mediastinal fistula

H500400 Empyema with pleural fistula NOS

H500500 Empyema with thoracic fistula NOS

H500z00 Empyema with fistula NOS

H501.00 Empyema with no fistula

H501000 Pleural abscess

H501100 Thorax abscess NOS

H501200 Pleural empyema

H501300 Lung empyema NOS

H501400 Purulent pleurisy

H501500 Pyopneumothorax

H501600 Pyothorax

H501z00 Empyema with no fistula NOS

H50z.00 Empyema NOS

H510900 Pneumococcal pleurisy

H510A00 Staphylococcal pleurisy

H510B00 Streptococcal pleurisy

H511.00 Bacterial pleurisy with effusion

H511000 Pneumococcal pleurisy with effusion

H511100 Staphylococcal pleurisy with effusion

H511200 Streptococcal pleurisy with effusion

H511z00 Bacterial pleurisy with effusion NOS

H51y.00 Other pleural effusion excluding mention of tuberculosis

H53..00 Abscess of lung and mediastinum

H530.00 Abscess of lung

H530000 Single lung abscess

H530100 Multiple lung abscess

H530200 Gangrenous pneumonia

H530300 Abscess of lung with pneumonia

H530z00 Abscess of lung NOS

H531.00 Abscess of mediastinum

H53z.00 Abscess of lung and mediastinum NOS

H56y100 Interstitial pneumonia

H57y500 Lung disease with syphilis

Hyu0.00 [X]Acute upper respiratory infections

Hyu0000 [X]Other acute sinusitis

Hyu0100 [X]Acute pharyngitis due to other specified organisms

Hyu0200 [X]Acute tonsillitis due to other specified organisms

Hyu0300 [X]Other acute upper respiratory infections/multiple sites

Hyu0400 [X]Flu+oth respiratory manifestations,'flu virus identified

Hyu0500 [X]Influenza+other manifestations,influenza virus identified

Hyu0600 [X]Influenza+oth respiratory manifestatns,virus not identifd

Hyu0700 [X]Influenza+other manifestations, virus not identified

Hyu0800 [X]Other viral pneumonia

Hyu0900 [X]Pneumonia due to other aerobic gram-negative bacteria

Hyu0A00 [X]Other bacterial pneumonia

Hyu0B00 [X]Pneumonia due to other specified infectious organisms

Hyu0C00 [X]Pneumonia in bacterial diseases classified elsewhere

Hyu0D00 [X]Pneumonia in viral diseases classified elsewhere

Hyu0E00 [X]Pneumonia in mycoses classified elsewhere

Hyu0F00 [X]Pneumonia in parasitic diseases classified elsewhere

Hyu0G00 [X]Pneumonia in other diseases classified elsewhere

Hyu0H00 [X]Other pneumonia, organism unspecified

Hyu1.00 [X]Other acute lower respiratory infections

Hyu1000 [X]Acute bronchitis due to other specified organisms

Hyu1100 [X]Acute bronchiolitis due to other specified organisms

L433000 Obstetric pyaemic and septic pulmonary embolism unspecified

L433100 Obstetric pyaemic and septic pulmonary embolism - delivered

L433200 Obstetric pyaemic and septic pulm embolism - deliv +p/n comp

L433300 Obstetric pyaemic and septic pulm embolism + a/n comp

L433400 Obstetric pyaemic and septic pulm embolism + p/n comp

L433z00 Obstetric pyaemic and septic pulmonary embolism NOS

R153000 [D]Positive culture findings in nose

R153100 [D]Positive culture findings in sputum

R153200 [D]Positive culture findings in throat

SP13200 Post operative chest infection

ab42.00 PULMONARY HISTOPLASMOSIS

h03..00 ACUTE TONSILLITIS

h060.00 ACUTE BRONCHITIS

2D64.00 O/E - purulent ear discharge

4JF1000 Ear swab culture positive

7303100 Drainage of abscess of external ear

A174.00 Tuberculosis of ear

A53x000 Herpes zoster otitis externa

A54x200 Herpes simplex otitis externa

A552.00 Postmeasles otitis media

A560200 Rubella deafness

F501111 Abscess, external ear

F501112 Cellulitis, external ear

F501200 Acute infection of pinna

F501300 Acute swimmers' ear

F501311 Beach ear

F501312 Tank ear

F501400 Infective otitis externa due to erysipelas

F501411 Erysipelas - otitis externa

F501500 Infective otitis externa due to herpes simplex

F501511 Herpes simplex- otitis externa

F501600 Infective otitis externa due to herpes zoster

F501611 Herpes zoster - otitis externa

F501700 Infective otitis externa due to impetigo

F501711 Impetigo - otitis externa

F501800 Furunculosis of external auditory meatus

F501900 Other acute external ear infections

F501A00 Malignant otitis externa

F501B00 Chronic otitis externa due to aspergillosis

F501C00 Chronic otitis externa due to moniliasis

F501D00 Chronic mycotic otitis externa NOS

F501E00 Other chronic infective otitis externa

F501F00 Chronic infective otitis externa NOS

F501G00 Haemorrhagic otitis externa

F501y00 Other specified infective otitis externa

F501z00 Infective otitis externa NOS

F506.00 Abscess of external ear

F510.00 Acute non suppurative otitis media

F510000 Acute otitis media with effusion

F510011 Acute secretory otitis media

F510100 Acute serous otitis media

F510200 Acute mucoid otitis media

F510300 Acute sanguinous otitis media

F510z00 Acute nonsuppurative otitis media NOS

F511.00 Chronic otitis media with effusion, serous

F511.11 Chronic secretory otitis media, serous

F511000 Chronic tubotympanic catarrh

F511100 Serosanguinous chronic otitis media

F511200 Bilateral chronic serous otitis

F511300 Unilateral chronic serous otitis

F511z00 Chronic serous otitis media NOS

F512.00 Chronic otitis media with effusion, mucoid

F512.11 Glue ear

F512.12 Chronic secretory otitis media, mucoid

F512000 Glue ear, unspecified

F512100 Mucosanguinous chronic otitis media

F512z00 Chronic mucoid otitis media NOS

F513.00 Chronic otitis media with effusion, other

F513100 Chronic otitis media with effusion, purulent

F513111 Chronic secretory otitis media, purulent

F513z00 Other chronic nonsuppurative otitis media NOS

F514.00 Unspecified nonsuppurative otitis media

F514100 Serous otitis media NOS

F514200 Catarrhal otitis media NOS

F514300 Mucoid otitis media NOS

F514z00 Nonsuppurative otitis media NOS

F515.00 Eustachian tube salpingitis

F515.11 Catarrh - eustachian

F515000 Unspecified eustachian tube salpingitis

F515100 Acute eustachian tube salpingitis

F515z00 Eustachian tube salpingitis NOS

F518.00 Chronic otitis media with effusion, unspecified

F52..00 Suppurative and unspecified otitis media

F520.00 Acute suppurative otitis media

F520000 Acute suppurative otitis media tympanic membrane intact

F520100 Acute suppurative otitis media tympanic membrane ruptured

F520300 Acute suppurative otitis media due to disease EC

F520z00 Acute suppurative otitis media NOS

F521.00 Chronic suppurative otitis media, tubotympanic

F522.00 Chronic suppurative otitis media, atticoantral

F523.00 Chronic suppurative otitis media NOS

F524.00 Purulent otitis media NOS

F524000 Bilateral suppurative otitis media

F525.00 Recurrent acute otitis media

F526.00 Acute left otitis media

F527.00 Acute right otitis media

F528.00 Acute bilateral otitis media

F52z.00 Otitis media NOS

F52z.11 Infection ear

F53..00 Mastoiditis and related conditions

F530.00 Acute mastoiditis

F530.12 Empyema of mastoid

F530000 Acute mastoiditis without complications

F530300 Acute mastoiditis with other complication

F530z00 Acute mastoiditis NOS

F531.00 Chronic mastoiditis

F531100 Post aural mastoid fistula

F531z00 Chronic mastoiditis NOS

F532.00 Petrositis

F532000 Unspecified petrositis

F532100 Acute petrositis

F532200 Chronic petrositis

F532z00 Petrositis NOS

F540.00 Acute myringitis without otitis media

F540100 Unspecified acute tympanitis

F540200 Bullous myringitis

F540z00 Acute myringitis NOS

F541.00 Chronic myringitis without mention of otitis media

F563.00 Labyrinthitis

F563000 Unspecified labyrinthitis

F563100 Serous labyrinthitis

F563200 Circumscribed labyrinthitis

F563300 Suppurative labyrinthitis

F563311 Purulent labyrinthitis

F563500 Viral labyrinthitis

F563z00 Labyrinthitis NOS

F586.00 Otorrhoea

F586000 Unspecified otorrhoea

F586011 Discharging ear NOS

F586z00 Otorrhoea NOS

FyuN000 [X]Other infective otitis externa

FyuN100 [X]Other otitis externa

FyuN300 [X]Otitis externa in bacterial diseases CE

FyuN400 [X]Otitis externa in viral diseases classified elsewhere

FyuN500 [X]Otitis externa in mycoses

FyuN600 [X]Otitis externa/other infectious+parasitic diseases CE

FyuP000 [X]Other acute nonsuppurative otitis media

FyuP100 [X]Other chronic nonsuppurative otitis media

FyuP200 [X]Other chronic suppurative otitis media

FyuP300 [X]Otitis media in bacterial diseases classified elsewhere

FyuP400 [X]Otitis media in viral diseases classified elsewhere

FyuP700 [X]Other mastoiditis and related conditions

FyuPD00 [X]Mastoiditis in infectious+parasitic diseases CE

FyuU400 [X]Acoustic neuritis in infectious+parasitic diseases CE

**Genitourinary tract infections**

ICPC Codes Description

U70 Pyelonephritis/pyelitis

U71 Cystitis/urinary infection other

U72 Urethritis

A70.10 RenalTuberculosis

A70.12 RenalTuberculosis

X70 Syphilis female

X71 Gonorrhoea female

X72 Genital candidiasis female

X73 Genital trichomoniasis female

X74 Pelvic inflammatory disease

X90 Genital herpes female

X91 Condylomata acuminata female

X92 Chlamydia infection genital (f)

Y70 Syphilis male

Y71 Gonorrhoea male

Y72 Genital herpes male

Y73 Prostatitis/seminal vesiculitis

Y74 Orchitis/epididymitis

Y75 Balanitis

Y76 Condylomata acuminata male

Read Codes Description

14D1.00 H/O: nephritis

14D2.00 H/O: kidney infection

14D4.00 H/O: recurrent cystitis

1592.00 H/O: pelvic infection

26A2.00 O/E - white vag. discharge

26A3.00 O/E - creamy vag. discharge

26A4.00 O/E - yellow vag. discharge

26A5.00 O/E - green vag. discharge

4382.00 Syphilis titre test positive

438B.00 Treponema pallidum ELISA positive

46B3.00 Urine bacteria test: positive

46G4.00 Urine micr.:leucocytes present

46G4.11 Leucocytes in urine

46G4.12 Sterile pyuria

46H..11 Bacteria in urine O/E

46H..14 Organism in urine O/E

46H..15 Parasite in urine

46H3.00 Urine micr.: parasites present

46H4.00 Urine micr.: bacteria present

46H6.00 Urine chlamydia trachomatis test positive

46U2.00 Urine culture - mixed growth

46U3.00 Urine culture - E. Coli

46U3.11 Urine culture - Escherich.coli

46U4.00 Urine culture - Proteus

46U5.00 Urine culture - Str. faecalis

46U6.00 Urine culture - Staph. albus

46U7.00 Urine culture - Pseudomonas

46U8.00 Urine culture - Bacteria OS

46U9.00 Urine cult - acid-fast bacilli

4963.00 Sperm: pyospermia O/E

4963.11 Pyospermia O/E

4JK1000 Urethral swab culture positive

4JK2000 High vaginal swab culture positive

4JK2200 HVS culture - trichomonas vaginalis

4JK2300 HVS culture - gardnerella vaginalis

4JK2400 High vaginal swab: fungal organism isolated

4JK5000 Cervical swab culture positive

4JK7.00 Vaginal swab culture positive

4JK8000 Penile swab culture positive

4K33.00 Cervical smear - trichomonas

4K34.00 Cervical smear - candida

4K35.00 Cerv.smear - viral infl.unsp.

4K35.11 Viral changes on cerv. smear

4K36.00 Cervical smear - wart virus

4K36.11 Herpes: cervical smear

4K36.12 HPV changes: cervical smear

4K37.00 Cervical smear - herpes

4K38.00 Cervical smear - actinomyces

4K39.00 Cervical smear - gardnerella

4K3A.00 Cervical smear: koilocytosis

4K3D.00 HPV - Human papillomavirus test positive

685P.00 HPV - Human papillomavirus test positive

7B37300 Aspiration of prostatic abscess

7B47000 Drainage of paravesical abscess

7E23400 Oophorotomy and drainage of abscess

7H21100 Open drainage of pelvic abscess

7H2A100 Image controlled percutaneous drainage of pelvic abscess

8H4A.00 Referred to venereologist

8HVP.00 Private referral to venereologist

9O8I.00 Smear infected - 1st recall

9O8J.00 Smear infected - 2nd recall

9O8K.00 Smear infected - 3rd recall

9O8L.00 Smear infected - recall delete

A05y100 Amoebic balanitis

A05z.00 Amoebiasis NOS

A16..00 Tuberculosis of genitourinary system

A160.00 Tuberculosis of kidney

A160.11 Renal tuberculosis

A160000 Tuberculous nephropathy

A160100 Tuberculous pyelitis

A160200 Tuberculous pyelonephritis

A160z00 Tuberculosis of kidney NOS

A161.00 Tuberculosis of bladder

A162.00 Tuberculosis of ureter

A163.00 Tuberculosis of other urinary organs

A164.00 Tuberculosis of epididymis

A165.00 Tuberculosis of other male genital organs

A165000 Tuberculosis of prostate

A165100 Tuberculosis seminal vesicle

A165200 Tuberculosis of testis

A165z00 Tuberculosis of other male genital organs NOS

A166.00 Tuberculous oophoritis or salpingitis

A166000 Tuberculous oophoritis

A166100 Tuberculous salpingitis

A166111 Fallopian tube tuberculosis

A166z00 Tuberculous oophoritis or salpingitis NOS

A167.00 Tuberculosis of other female genital organs

A167000 Tuberculous cervicitis

A167100 Tuberculous endometritis

A167z00 Tuberculosis of other female genital organs NOS

A168.00 Tuberculosis of urinary tract

A16z.00 Genitourinary tuberculosis NOS

A32y300 Diphtheritic cystitis

A32y500 Diphtheria of penis

A3By200 Mima polymorpha infection

A3By700 Gardnerella vaginalis

A541.00 Genital herpes simplex

A541000 Genital herpes unspecified

A541100 Herpetic vulvovaginitis

A541200 Herpetic ulceration of vulva

A541300 Herpetic infection of penis

A541400 Herpesviral infection of perianal skin and rectum

A541500 Anogenital herpesviral infection

A541z00 Genital herpes simplex NOS

A720.00 Mumps orchitis

A781200 Genital warts

A781211 Condylomata acuminatum

A781212 Penile warts

A781213 Venereal warts

A781300 Perianal warts

A781311 Anal warts

A786.00 Haemorrhagic nephrosonephritis

A78A000 Chlamydial infection of lower genitourinary tract

A78A300 Chlamydial inf of pelviperitoneum oth genitourinary organs

A78AX00 Chlamydial infection of genitourinary tract, unspecified

A844100 Plasmodium malariae malaria with nephropathy

A9...00 Syphilis and other venereal diseases

A9...11 Sexually transmitted diseases

A9...12 Venereal diseases

A900.00 Early congenital syphilis with symptoms

A91..00 Early symptomatic syphilis

A910.00 Primary genital syphilis

A910.11 Genital chancre

A911.00 Primary anal syphilis

A913500 Secondary syphilis of vulva

A954.00 Syphilis of kidney

A954.11 Renal syphilis

A98..00 Gonococcal infections

A980.00 Acute gonorrhoea of lower genitourinary tract

A980000 Acute gonococcal Bartholinitis

A980100 Acute gonococcal urethritis

A980200 Acute gonococcal vulvovaginitis

A980z00 Acute gonorrhoea of lower genitourinary tract NOS

A981.00 Acute gonorrhoea of upper genitourinary tract

A981000 Acute unspecified gonorrhoea of upper genitourinary tract

A981100 Acute gonococcal cystitis

A981111 Bladder gonorrhoea - acute

A981200 Acute gonococcal prostatitis

A981300 Acute gonococcal epididymo-orchitis

A981311 Acute gonococcal orchitis

A981400 Acute gonococcal seminal vesiculitis

A981500 Acute gonococcal cervicitis

A981600 Acute gonococcal endometritis

A981611 Uterus - acute gonorrhoea

A981700 Acute gonococcal salpingitis

A981z00 Acute gonorrhoea upper genitourinary tract NOS

A982.00 Chronic gonorrhoea lower genitourinary tract

A982000 Chronic gonococcal bartholinitis

A982100 Chronic gonococcal urethritis

A982200 Chronic gonococcal vulvovaginitis

A982z00 Chronic gonorrhoea of lower genitourinary tract NOS

A983.00 Chronic gonorrhoea of upper genitourinary tract

A983000 Chronic unspecified gonorrhoea of upper genitourinary tract

A983100 Chronic gonococcal cystitis

A983200 Chronic gonococcal prostatitis

A983300 Chronic gonococcal epididymo-orchitis

A983400 Chronic gonococcal seminal vesiculitis

A983500 Chronic gonococcal cervicitis

A983600 Chronic gonococcal endometritis

A983611 Uterus - chronic gonorrhoea

A983700 Chronic gonococcal salpingitis

A983z00 Chronic gonorrhoea of upper genitourinary tract NOS

A98z.11 Gonorrhoea

A99..00 Other venereal diseases

A994.00 Nonspecific urethritis

AC2z000 Echinococcosis kidney

AD10.00 Urogenital trichomonas

AD10000 Unspecified urogenital trichomonas

AD10011 Flour vaginalis - trichomonal

AD10012 Leukorrhoea vaginalis - trichomonal

AD10100 Trichomonal vulvovaginitis

AD10111 Trichomonal vaginitis

AD10200 Trichomonal urethritis

AD10300 Trichomonal prostatitis

AD10z00 Urogenital trichomonas NOS

AE02.00 Late effects of genitourinary system tuberculosis

Ayu4G00 [X]Anogenital herpes viral infection, unspecified

Ayu4K00 [X]Chlamydial infection of genitourinary tract, unspecified

AyuJ100 [X]Sequelae of genitourinary tuberculosis

K00..00 Acute glomerulonephritis

K00..11 Acute nephritis

K000.00 Acute proliferative glomerulonephritis

K001.00 Acute nephritis with lesions of necrotising glomerulitis

K00y.00 Other acute glomerulonephritis

K00y000 Acute glomerulonephritis in diseases EC

K00y100 Acute exudative nephritis

K00y200 Acute focal nephritis

K00y300 Acute diffuse nephritis

K00yz00 Other acute glomerulonephritis NOS

K00z.00 Acute glomerulonephritis NOS

K01x200 Nephrotic syndrome in malaria

K08y500 Acute interstitial nephritis

K0B0.00 Ren tubulo-interstital disord infect and parasitic dis EC

K0y0.00 Late syphilis of kidney

K10..00 Infections of kidney

K10..11 Renal infections

K100.00 Chronic pyelonephritis

K100000 Chronic pyelonephritis without medullary necrosis

K100100 Chronic pyelonephritis with medullary necrosis

K100200 Chronic pyelitis

K100300 Chronic pyonephrosis

K100400 Nonobstructive reflux-associated chronic pyelonephritis

K100500 Chronic obstructive pyelonephritis

K100z00 Chronic pyelonephritis NOS

K101.00 Acute pyelonephritis

K101000 Acute pyelonephritis without medullary necrosis

K101100 Acute pyelonephritis with medullary necrosis

K101200 Acute pyelitis

K101300 Acute pyonephrosis

K101z00 Acute pyelonephritis NOS

K102.00 Renal and perinephric abscess

K102000 Renal abscess

K102100 Perinephric abscess

K102200 Renal carbuncle

K102z00 Renal and perinephric abscess NOS

K103.00 Pyeloureteritis cystica

K103.11 Ureteritis cystica

K103.12 Infestation of renal pelvis with ureter

K104.00 Xanthogranulomatous pyelonephritis

K10y.00 Pyelonephritis and pyonephrosis unspecified

K10y000 Pyelonephritis unspecified

K10y100 Pyelitis unspecified

K10y200 Pyonephrosis unspecified

K10y300 Pyelonephritis in diseases EC

K10y400 Pyelitis in diseases EC

K10yz00 Unspecified pyelonephritis NOS

K10z.00 Infection of kidney NOS

K13y300 Periureteritis

K15..00 Cystitis

K150.00 Acute cystitis

K152.00 Other chronic cystitis

K152000 Subacute cystitis

K152y00 Chronic cystitis unspecified

K152z00 Other chronic cystitis NOS

K153.00 Trigonitis

K153.11 Follicular cystitis

K153000 Acute trigonitis

K153200 Urethrotrigonitis

K153z00 Trigonitis NOS

K154.00 Cystitis in diseases EC

K154000 Cystitis in actinomycosis

K154100 Cystitis in amoebiasis

K154200 Cystitis in bilharziasis

K154300 Cystitis in echinococcus infestation

K154400 Cystitis in diphtheria

K154500 Cystitis in gonorrhoea

K154600 Cystitis in moniliasis

K154700 Cystitis in trichomoniasis

K154800 Cystitis in tuberculosis

K154z00 Cystitis in diseases EC NOS

K155.00 Recurrent cystitis

K15y.00 Other specified cystitis

K15y200 Abscess of bladder

K15yz00 Other cystitis NOS

K15z.00 Cystitis NOS

K17..11 Periurethritis

K170.00 Urethral and periurethral abscess

K170.11 Urethral abscess

K170000 Urethral abscess unspecified

K170300 Periurethral cellulitis

K170311 Periurethritis

K170400 Periurethral abscess

K170z00 Urethral abscess NOS

K172.00 Candidal urethritis

K17y.00 Other urethritis

K17y000 Urethritis unspecified

K17y400 Urethral meatitis

K17y500 Urethral meatal ulcer

K17y600 Verumontanitis

K17y700 Utriculus masculinus

K17yz00 Other urethritis NOS

K17z.00 Urethritis due to non venereal cause NOS

K180.00 Infective urethral stricture

K180100 Urethral stricture due to infection EC

K180z00 Infective urethral stricture NOS

K190.00 Urinary tract infection, site not specified

K190.11 Recurrent urinary tract infection

K190000 Bacteriuria, site not specified

K190011 Asymptomatic bacteriuria

K190100 Pyuria, site not specified

K190200 Post operative urinary tract infection

K190300 Recurrent urinary tract infection

K190311 Recurrent UTI

K190400 Chronic urinary tract infection

K190z00 Urinary tract infection, site not specified NOS

K1A..00 Urinary calculus in schistosomiasis

K210.00 Acute prostatitis

K212.00 Abscess of prostate

K213.00 Prostatocystitis

K214.00 Prostatitis in diseases EC

K214000 Prostatitis in actinomycosis

K214100 Prostatitis in blastomycosis

K214200 Prostatitis in syphilis

K214300 Prostatitis in tuberculosis

K214400 Prostatitis in gonorrhoea

K214500 Prostatitis in moniliasis

K214600 Prostatitis in trichomoniasis

K214z00 Prostatitis in diseases EC NOS

K21z.00 Prostatitis NOS

K24..00 Orchitis and epididymitis

K240.00 Orchitis

K240000 Orchitis with abscess

K240100 Orchitis with no abscess

K240200 Orchitis unspecified

K240300 Orchitis in diseases EC

K240z00 Orchitis NOS

K241.00 Epididymitis

K241000 Epididymitis with abscess

K241100 Epididymitis with no abscess

K241200 Epididymitis unspecified

K241300 Epididymitis in diseases EC

K241400 Acute epididymitis

K241500 Chronic epididymitis

K241600 Chlamydial epididymitis

K241z00 Epididymitis NOS

K242.00 Epididymo-orchitis

K242000 Epididymo-orchitis with abscess

K242100 Epididymo-orchitis with no abscess

K242200 Epididymo-orchitis unspecified

K242300 Epididymo-orchitis in diseases EC

K242z00 Epididymo-orchitis NOS

K24z.00 Orchitis and epididymitis NOS

K26y100 Infertility due to infective cause

K271.00 Balanoposthitis

K271.11 Balanitis

K271000 Balanitis

K271100 Posthitis

K271200 Zoon's balanitis

K271z00 Balanoposthitis NOS

K272.00 Other penile inflammatory disorders

K272.11 Infection of penis

K272000 Penile abscess

K272100 Penile boil

K272200 Penile carbuncle

K272300 Cellulitis of penis

K272z00 Other penile inflammatory disorder NOS

K27y500 Chronic ulcer of penis

K280.00 Seminal vesiculitis

K280000 Seminal vesiculitis unspecified

K280100 Abscess of seminal vesicle

K280200 Cellulitis of seminal vesicle

K280z00 Seminal vesiculitis NOS

K284000 Abscess of scrotum

K284100 Boil of scrotum

K284200 Carbuncle of scrotum

K284300 Cellulitis of scrotum

K284400 Abscess of spermatic cord

K284500 Vasitis

K28y300 Ulcer of scrotum

K310.11 Abscess, breast, non puerperal

K310.12 Mastitis - non puerperal

K310.13 Mastitis

K310000 Acute nonpuerperal mastitis

K310100 Acute adolescent mastitis

K310111 Pubertal mastitis

K310200 Subacute nonpuerperal mastitis

K310300 Chronic nonpuerperal mastitis

K310400 Acute nonpuerperal breast abscess

K310500 Chronic nonpuerperal breast abscess

K310600 Chronic subareolar nonpuerperal abscess

K310700 Retromammary breast abscess

K310800 Breast infection

K310900 Nonpuerperal breast fistula

K318.00 Breast abscess

K400.11 Oophoritis - acute

K400000 Acute oophoritis

K400100 Acute perioophoritis

K400200 Acute salpingo-oophoritis

K400300 Acute salpingitis

K400400 Acute perisalpingitis

K400500 Subacute oophoritis

K400600 Subacute perioophoritis

K400700 Subacute salpingo-oophoritis

K400800 Subacute salpingitis

K400900 Subacute perisalpingitis

K400z00 Acute salpingitis and oophoritis NOS

K401.00 Chronic salpingitis and oophoritis

K401000 Chronic oophoritis

K401100 Chronic perioophoritis

K401200 Chronic salpingo-oophoritis

K401300 Chronic salpingitis

K401400 Chronic perisalpingitis

K401500 Hydrosalpinx

K401600 Salpingitis follicularis

K401700 Salpingitis isthmica nodosa

K401z00 Chronic salpingitis and oophoritis NOS

K402.00 Salpingitis and oophoritis unspecified

K402000 Fallopian tube abscess

K402011 Pyosalpinx

K402100 Ovarian abscess

K402200 Tubo-ovarian abscess

K402300 Oophoritis unspecified

K402400 Perioophoritis unspecified

K402500 Salpingo-oophoritis unspecified

K402600 Salpingitis unspecified

K402700 Perisalpingitis unspecified

K402z00 Unspecified salpingitis and oophoritis NOS

K403.00 Acute parametritis and pelvic cellulitis

K403000 Acute parametritis

K403100 Acute pelvic cellulitis

K403111 Acute pelvic abscess - female

K403z00 Acute parametritis and pelvic cellulitis NOS

K404.00 Chronic parametritis and pelvic cellulitis

K404000 Chronic female pelvic cellulitis

K404100 Chronic abscess of the broad ligament

K404200 Chronic abscess of the parametrium

K404300 Chronic abscess of the female pelvis

K404400 Chronic abscess of the pouch of Douglas

K404500 Female chronic pelvic peritonitis

K404z00 Chronic pelvic inflammatory diseases NOS

K405.00 Parametritis and pelvic cellulitis unspecified

K405000 Parametritis unspecified

K405100 Pelvic cellulitis unspecified

K405z00 Parametritis and pelvic cellulitis NOS

K406.00 Acute and unspecified female pelvic peritonitis

K406.11 Acute pelvic inflammatory disease

K406000 Acute female pelvic peritonitis

K406100 Female pelvic peritonitis unspecified

K406z00 Acute and unspecified female pelvic peritonitis NOS

K407.00 Female pelvic peritoneal adhesions

K407000 Peritubal peritoneal adhesions

K407100 Tubo-ovarian peritoneal adhesions

K407z00 Female pelvic peritoneal adhesions NOS

K408.00 Other chronic female pelvic peritonitis

K409.00 Acute pelvic inflammatory disease

K40y.00 Other female pelvic organ inflammatory diseases

K40y000 Female syphilitic pelvic inflammatory disease

K40y100 Female chlamydial pelvic inflammatory disease

K40z.00 Female pelvic inflammatory diseases NOS

K40z.11 PID

K40z.12 Female pelvic infection

K40z.13 PID - pelvic inflammatory disease

K41..00 Uterine inflammatory diseases excluding the cervix

K410.00 Acute uterine inflammatory disease

K410000 Acute endometritis

K410100 Acute endomyometritis

K410200 Acute myometritis

K410300 Acute perimetritis

K410400 Acute pyometra or uterine abscess

K410500 Subacute endometritis

K410600 Subacute endomyometritis

K410700 Subacute myometritis

K410800 Subacute perimetritis

K410900 Subacute pyometra

K410z00 Acute uterine inflammatory diseases NOS

K411.00 Chronic uterine inflammatory disease

K411000 Chronic endometritis

K411100 Chronic endomyometritis

K411200 Chronic myometritis

K411300 Chronic perimetritis

K411400 Chronic pyometra

K411z00 Chronic uterine inflammatory disease NOS

K41z.00 Unspecified uterine inflammatory disease

K41z000 Endometritis unspecified

K41z100 Endomyometritis unspecified

K41z200 Myometritis unspecified

K41z300 Perimetritis unspecified

K41z400 Pyometra unspecified

K41zz00 Unspecified uterine inflammatory disease NOS

K42..00 Cervical, vaginal and vulval inflammatory diseases

K420.00 Cervicitis and endocervicitis

K420000 Cervicitis unspecified

K420100 Endocervicitis unspecified

K420200 Acute cervicitis

K420300 Cervicitis with erosion

K420400 Cervicitis with Nabothian cyst

K420500 Cervicitis with ectropion

K420600 Endocervicitis with erosion

K420700 Endocervicitis with Nabothian cyst

K420800 Endocervicitis with ectropion

K420900 Chlamydia cervicitis

K420A00 Nabothian follicles

K420A11 Nabothian cyst

K420B00 Chronic cervicitis

K420z00 Cervicitis and endocervicitis NOS

K420z11 Nabothian follicles NOS

K421.00 Vaginitis and vulvovaginitis

K421000 Vaginitis unspecified

K421100 Vulvitis unspecified

K421111 Vulval sores

K421200 Vulvovaginitis unspecified

K421400 Vaginitis in diseases EC

K421500 Vulvitis in diseases EC

K421600 Vulvovaginitis in diseases EC

K421700 Subacute and chronic vaginitis

K421800 Subacute and chronic vulvitis

K421900 Bacterial vaginitis

K421911 Bacterial vaginosis

K421A00 Acute vulvitis

K421z00 Vaginitis and vulvovaginitis NOS

K424.00 Other abscess of vulva

K424000 Abscess of vulva

K424011 Abscess of labia

K424100 Carbuncle of vulva

K424111 Boil of vulva

K424z00 Other abscess of vulva NOS

K425.00 Ulceration of vulva

K425000 Ulceration of vulva unspecified

K425100 Ulceration of vulva in diseases EC

K425z00 Ulceration of vulva NOS

K42y000 Carbuncle of vagina

K42y200 Ulcer of vagina

K42z.00 Cervical, vaginal and vulval inflammatory disease NOS

K43..00 Female tuberculous pelvic inflammatory disease

K44..00 Female gonococcal pelvic inflammatory disease

K4y..00 Other specified female pelvic inflammatory disease

K4z..00 Female pelvic inflammatory disease NOS

Kyu0000 [X]Glomerular disorders in infectious+parasitic diseases CE

Kyu1700 [X]Renal tubulo-interstitial disordr/infect+parasitic dis CE

Kyu4200 [X]Oth disordrs/kidney+ureter/infects+parasitic diseases CE

Kyu5000 [X]Other chronic cystitis

Kyu5100 [X]Other cystitis

Kyu5500 [X]Other urethritis

Kyu6A00 [X]Balanitis in diseases classified elsewhere

Kyu8400 [X]Ulceration of vulva in infectious+parasitic diseases CE

Kyu8500 [X]Vaginits,vulvits+vulvovaginitis/infect+parasitc diseas CE

Kyu8600 [X]Vulvovaginal ulceration+inflammation in other diseases CE

Kyu8700 [X]Female pelvic peritonitis, unspecified

L040000 Unspec spontaneous abortion + genital tract/pelvic infection

L041000 Incomp spontaneous abortion + genital tract/pelvic infection

L042000 Complete spontaneous abortion + genital tract/pelvic infect

L043000 Unspec inev abor comp by genital tract and pelvic infect

L043011 Unspec inev miscarriage comp by genital tract pelvic infec

L044000 Incomp inev abor comp by genital tract and pelvic infection

L044011 Incomp inev mis complicated by genital tract pelvic infect

L045000 Complete inev abor comp by genital tract and pelvic infec

L045011 Complete inev misc compl by genital tract and pelvic infec

L050000 Unspecified legal abortion + genital tract/pelvic infection

L051000 Incomplete legal abortion + genital tract/pelvic infection

L052000 Complete legal abortion + genital tract or pelvic infection

L060000 Unspec illegal abortion + genital tract or pelvic infection

L061000 Incomplete illegal abortion + genital tract/pelvic infection

L062000 Complete illegal abortion + genital tract/pelvic infection

L070000 Unspecified abortion with genital tract or pelvic infection

L071000 Unspecified incomplete abortion +genital tract/pelvic infect

L072000 Unspecified complete abortion + genital tract/pelvic infect

L080.00 Failed attempted abortion + genital tract/pelvic infection

L090.00 Genital or pelvic infection following abortive pregnancy

L090000 Endometritis following abortive pregnancy

L090100 Parametritis following abortive pregnancy

L090200 Pelvic peritonitis following abortive pregnancy

L090300 Salpingitis following abortive pregnancy

L090400 Salpingo-oophoritis following abortive pregnancy

L09y400 Urinary tract infection following abortive pregnancy

L0A1.00 Failed medical abortion complic by genital tract/pelvic infn

L165.00 Asymptomatic bacteriuria in pregnancy

L165000 Asymptomatic bacteriuria in pregnancy unspecified

L165100 Asymptomatic bacteriuria in pregnancy - delivered

L165200 Asymptomatic bacteriuria in pregnancy - del with p/n comp

L165300 Asymptomatic bacteriuria in pregnancy - not delivered

L165400 Asymptomatic bacteriuria in pregnancy with postnatal comp

L165z00 Asymptomatic bacteriuria in pregnancy NOS

L166.00 Genitourinary tract infections in pregnancy

L166.11 Cystitis of pregnancy

L166000 Genitourinary tract infection in pregnancy unspecified

L166100 Genitourinary tract infection in pregnancy - delivered

L166200 Genitourinary tract infection in pregnancy - deliv +p/n comp

L166300 Genitourinary tract infection in pregnancy - not delivered

L166400 Genitourinary tract infection in pregnancy with p/n comp

L166500 Infections of kidney in pregnancy

L166600 Urinary tract infection following delivery

L166700 Infections of the genital tract in pregnancy

L166800 Urinary tract infection complicating pregnancy

L166z00 Genitourinary tract infection in pregnancy NOS

L166z11 UTI - urinary tract infection in pregnancy

L177.00 Infections of bladder in pregnancy

L178.00 Infections of urethra in pregnancy

L284.00 Amniotic cavity infection

L284.11 Amnionitis

L284.12 Chorioamnionitis

L284.13 Membranitis

L284.14 Placentitis

L284000 Amniotic cavity infection unspecified

L284100 Amniotic cavity infection - delivered

L284200 Amniotic cavity infection with antenatal problem

L284z00 Amniotic cavity infection NOS

L28y.00 Other problems of amniotic cavity and membranes

L400.00 Puerperal endometritis

L400000 Puerperal endometritis unspecified

L400100 Puerperal endometritis - delivered with postnatal comp

L400200 Puerperal endometritis with postnatal complication

L400z00 Puerperal endometritis NOS

L401.00 Puerperal salpingitis

L401000 Puerperal salpingitis unspecified

L401100 Puerperal salpingitis - delivered with postnatal comp

L401200 Puerperal salpingitis with postnatal complication

L401z00 Puerperal salpingitis NOS

L45..00 Obstetric breast infections

L450.00 Obstetric nipple infection

L450.11 Abscess of nipple - obstetric

L450.12 Nipple infection - obstetric

L450000 Obstetric nipple infection unspecified

L450100 Obstetric nipple infection - delivered

L450200 Obstetric nipple infection - delivered with p/n complication

L450300 Obstetric nipple infection with antenatal complication

L450400 Obstetric nipple infection with postnatal complication

L450z00 Obstetric nipple infection NOS

L451.00 Obstetric breast abscess

L451.11 Purulent mastitis - obstetric

L451000 Obstetric breast abscess unspecified

L451100 Obstetric breast abscess - delivered

L451200 Obstetric breast abscess - deliv with postnatal complication

L451300 Obstetric breast abscess with antenatal complication

L451400 Obstetric breast abscess with postnatal complication

L451z00 Obstetric breast abscess NOS

L452.00 Obstetric nonpurulent mastitis

L452000 Obstetric nonpurulent mastitis unspecified

L452100 Obstetric nonpurulent mastitis - delivered

L452200 Obstetric nonpurulent mastitis - deliv with p/n complication

L452300 Obstetric nonpurulent mastitis with antenatal complication

L452400 Obstetric nonpurulent mastitis with postnatal complication

L452z00 Obstetric nonpurulent mastitis NOS

L45y.00 Other obstetric breast infections

L45y000 Other obstetric breast infection unspecified

L45y100 Other obstetric breast infection - delivered

L45y200 Other obstetric breast infection - deliv with p/n comp

L45y300 Other obstetric breast infection with antenatal complication

L45y400 Other obstetric breast infection with postnatal complication

L45yz00 Other obstetric breast infection NOS

L45z.00 Obstetric breast infection NOS

L45z000 Obstetric breast infection NOS, unspecified

L45z100 Obstetric breast infection NOS - delivered

L45z200 Obstetric breast infection NOS - deliv with p/n complication

L45z300 Obstetric breast infection NOS with antenatal complication

L45z400 Obstetric breast infection NOS with postnatal complication

L45zz00 Obstetric breast infection NOS

Lyu0400 [X]Oth+unspcf failed inducd abort,complct gen tract+pelv inf

Lyu2300 [X]Infections of other parts of urinary tract in pregnancy

Lyu2400 [X]Other+unspcf genitourinary tract infection in pregnancy

Lyu6000 [X]Other infection of genital tract following delivery

Lyu6100 [X]Other genitourinary tract infections following delivery

Lyu6B00 [X]Vaginitis following delivery

Lyu6C00 [X]Cervicitis following delivery

R118.00 [D]Abnormal findings on microbiological exam of urine

R12C500 [D]Abnrm microbiolog find in specim/female genital organs

R150100 [D] Koilocytosis - cervical smear

SP07700 Infect+inflam react due pros dev,implt+graft in urinary syst

SP07800 Infect+inflam react due/prosth dev,implant+graft in gen trct

Z262J00 Placenta infected

ab21.00 Candidal Vulvovaginitis

K420b00 Chronic Cervicitis

**Other infections**

ICPC Codes Description

A70 Tuberculosis ( NO RESP)

A71 Measles

A72 Chickenpox

A73 Malaria

A74 Rubella

A75 Infectious mononucleosis

A76 Viral exanthem other

A77 Viral disease other/NOS

A78 Infectious disease other/NOS

B70 Lymphadenitis acute

B71 Lymphadenitis non-specific

D70 Gastrointestinal infection

D71 Mumps

D72 Viral hepatitis

D73 Gastroenteritis presumed infection

F70 Conjunctivitis infectious

F71 Conjunctivitis allergic

F72 Blepharitis/stye/chalazion

F73 Eye infection/inflammation other

K70 Infection of circulatory system

K71 Rheumatic fever/heart disease

L70 Infections musculoskeletal system

N70 Poliomyelitis

N71 Meningitis/encephalitis

N72 Tetanus

N73 Neurological infection other

S76 Skin infection other

S84 Impetigo

T70 Endocrine infection

W70 Puerperal infection/sepsis

W71 Infection complicating pregnancy

Read Codes Description

A....00: Az...00 Infectious and parasitic diseases (Excluding above groups)

Infectious disease under the other READ headings
